# Spatial development of transport structures in apple (*Malus* × *domestica* Borkh.) fruit

**DOI:** 10.3389/fpls.2015.00679

**Published:** 2015-09-01

**Authors:** Els Herremans, Pieter Verboven, Maarten L. A. T. M. Hertog, Dennis Cantre, Mattias van Dael, Thomas De Schryver, Luc Van Hoorebeke, Bart M. Nicolaï

**Affiliations:** ^1^Division of MeBioS, Department of Biosystems, KU Leuven, University of LeuvenLeuven, Belgium; ^2^Department of Physics and Astronomy, UGCT-Radiation Physics, Ghent UniversityGhent, Belgium; ^3^Flanders Centre of Postharvest TechnologyLeuven, Belgium

**Keywords:** growth model, microtomography, gas and water transport, vascular system, programmed cell death

## Abstract

The void network and vascular system are important pathways for the transport of gases, water and solutes in apple fruit (*Malus* × *domestica* Borkh). Here we used X-ray micro-tomography at various spatial resolutions to investigate the growth of these transport structures in 3D during fruit development of “Jonagold” apple. The size of the void space and porosity in the cortex tissue increased considerably. In the core tissue, the porosity was consistently lower, and seemed to decrease toward the end of the maturation period. The voids in the core were more narrow and fragmented than the voids in the cortex. Both the void network in the core and in the cortex changed significantly in terms of void morphology. An automated segmentation protocol underestimated the total vasculature length by 9–12% in comparison to manually processed images. Vascular networks increased in length from a total of 5 m at 9 weeks after full bloom, to more than 20 m corresponding to 5 cm of vascular tissue per cubic centimeter of apple tissue. A high degree of branching in both the void network and vascular system and a complex three-dimensional pattern was observed across the whole fruit. The 3D visualizations of the transport structures may be useful for numerical modeling of organ growth and transport processes in fruit.

## Introduction

Transport processes play a vital role in the physiology of apple (*Malus* × *domestica* Borkh) fruit. Gas transport includes the delivery of oxygen from the atmosphere toward the mitochondria, removal of respiratory carbon dioxide, and fermentative metabolites such as ethanol in the opposite direction, and distribution of gaseous plant hormones such as ethylene. Water transport drives cell expansion, and supplies minerals and sugars toward to cells. Fruit have many structural features at various spatial scales such as stomata, lenticels, plasmodesmata, and aquaporins that facilitate transport processes; yet the intercellular space and vascular system provide the main route for bulk transport of gases and water, respectively.

**Figure 1:**
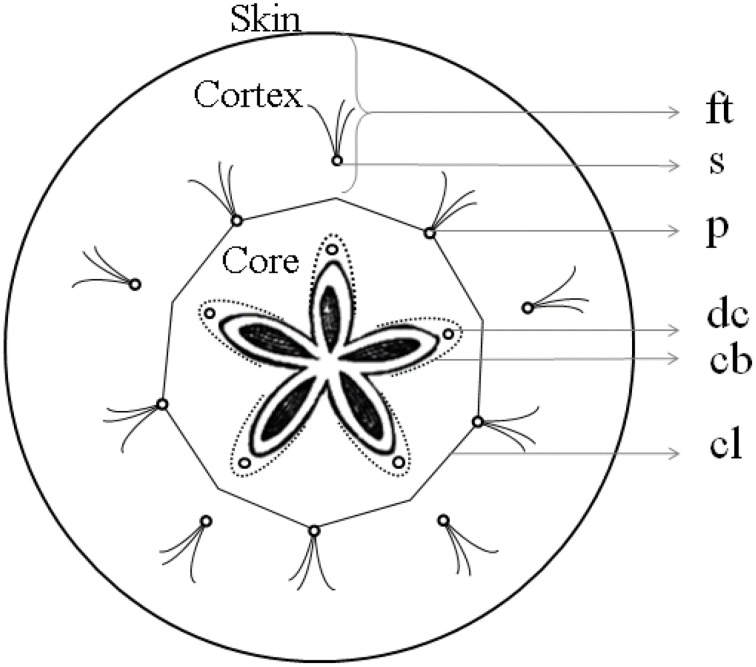
**Diagram of apple fruit in transverse section showing gross morphology of apple fruit**. ft, floral tube; s, sepal bundles; p, petal bundles; cl, outer limit of carpel or core line; dc, dorsal carpellary bundle; cb, carpellary bundles connecting dorsal with ventral carpellary bundles (Based on MacDaniels, 1940).

The intercellular space is the continuum consisting of all voids or pores between the cells and may encompass as much as 30% of the total volume of an apple fruit depending on the cultivar (Mebatsion et al., 2009). From the center to the periphery of the apple cortex, the porosity due to the intercellular spaces increases gradually. The epidermis, in contrast, consists of highly connected cells with little air spaces in between. The void arrangement differs between different apple cultivars (Herremans et al., 2013b) and the void volume increases during apple growth (Mendoza et al., 2010). The intercellular space contains air, depleted of O_2_ to various degrees and enriched in CO_2_, as well as other metabolic gases, and is close to saturated with water vapor (Kader, 2002). The vascular system consists of bundles of xylem and phloem that are responsible for transport of water loaded with minerals and transport sugars, respectively. Vascular bundles are arranged in two distinct systems: “cortical” and “carpellary” with respect to their position within the fruit, in the cortex or in the core (Figure 1). The cortical vascular system has 10 primary (sepal and petal) bundles that bend around the carpels, extending into a network of vessels that ramify toward the skin. The carpellary vascular system is composed of 10 ventral and five dorsal bundles that merge with each other. The paired ventral bundles emerge from the stele after the separation of primary bundles to form a ring along the ventral lobes of the carpels, with branching lateral traces to the ovules. Dorsal bundles diverge from the primary (sepal) bundles, swinging around the locules, and terminating together with the ventral bundles in the fused styles of the pistil. The conducting cells of the xylem are lignified, providing good mechanical support. This is, however, at the expense of elasticity of these transport pathways that are subject to stress during growth (Drazeta et al., 2004b). Multiple xylem vessels are joined together and surrounded by phloem tissue, which is composed of living conducting elements, without intercellular spaces, and that in general can tolerate more strain (Verboven et al., 2008). Venetian patterns are considered characteristic to a level that allows them to be used for phylogenetic traits, but phenotypic plasticity, even between individual organs of the same plant, exists (Mattsson et al., 1999; Wenzel et al., 2012), the mechanisms for such response being considerably complex (Wenzel et al., 2012).

Understanding the origin and design of a plant's transport system is imperative to explain the way plants connect new tissue “hardware” as they grow, while maintaining control of their internal resources (Pickard and Melcher, 2005). Traditionally, histological studies have been performed to gain insight in plant and tissue anatomy (MacDaniels, 1940; Tukey and Young, 1942). However, such studies provide only 2D information, while transport processes in the tissues are essentially 3D (Ho et al., 2011). New tomographic imaging methods enable a thorough update of the blueprints of these long known transport pathways and how they develop during fruit growth.

X-ray micro-Computed Tomography (μCT) has been employed to visualize and characterize voids in a variety of fleshy fruit tissue samples such as cucumber (Kuroki et al., 2004), pome fruit (Mendoza et al., 2007; Verboven et al., 2008), mango (Cantre et al., 2014b), and kiwi (Cantre et al., 2014a). X-ray μCT is likely the most appropriate technique to study voids in 3D as it provides a relatively large contrast between air and cells even for high resolutions (as low as 1 μm and beyond). Also, either intact samples (entire fruit) or excised but alive samples can be visualized without further preprocessing. The processes underlying the development of voids in apple are yet unclear, but the shape of the voids in mature apple fruit suggests a lysigenous origin (Verboven et al., 2008). The void network of developing apple fruit, and in a broader context, void formation, has not been studied using X-ray micro-CT, though. MRI (Magnetic Resonance Imaging) has been applied to visualize the vasculature of sugar beet, apple, fig, okra pod, kiwi, potato (MacFall and Johnson, 1994), and developing blackcurrant fruit (Glidewell et al., 1999). These studies, however, were limited to visualizations of the vascular bundles in 2D. The same is true for the study of leaf venation networks that were imaged by means of X-ray radiography (Blonder et al., 2012) and neutron tomography (Defraeye et al., 2014). An actual 3D reconstruction of a vessel network was achieved by Brodersen et al. (2011) based on synchrotron X-ray CT images of grapevine stems (McElrone et al., 2013). To our knowledge, the vasculature of a fleshy fruit, such as developing apple fruit, has not been investigated in quantitative detail yet. Only recently, the volume of vascular tissue was assessed as a function of the growing process (Moriwaki et al., 2014).

This study aims to provide an insight on the changes in the intercellular space and vascular system of apple during fruit development, as well as describe quantitatively their three-dimensional structure in the fruit as a basis for improved modeling of important processes such as gas, water and nutrient transport. This was achieved through X-ray micro-CT of the whole fruit at distinct stages during fruit development combined with advanced image processing techniques.

## Materials and methods

### Apple fruit

Four “Jonagold” apple trees (*Malus* × *domestica* Borkh.) bearing a large amount of flower buds were selected on 23 April 2013 in an orchard of the experimental station “Fruitteeltcentrum” in Rillaar (Belgium). The trees were 6 years old, and positioned next to each other in a tree line. Starting from 2 May 2013, fruit were harvested at regular intervals, the first 40 days sampling occurred weekly, later at larger intervals of about 2 weeks, up to 24 September 2013 (week 22 after full bloom). Developing apple clusters or apples were harvested in the morning (between 08:00 and 09:00 h) at eye level, on the east side of the tree, while ensuring that the most developed fruit was picked. Harvested fruit were weighed on a digital scale and the equatorial diameter was measured by means of a digital caliper. Starting from week 12, the firmness of the apples was determined by means of a penetration test on a universal texture analyser (LRX, Lloyd Instruments, UK) with a self-cutting cylindrical plunger with a diameter of 11.3 mm and a constant speed of 8 mm s^−1^. The measurements were taken on the equator, at the blush side of 20 apples, and the average was taken as the firmness value.

### X-ray micro-CT

Apples were harvested at different weeks after full bloom. One day after harvest, apples were scanned by means of X-ray micro-CT. Two types of scans were performed on two different X-ray micro-CT devices. High-resolution scans of excised tissue samples were obtained on a SkyScan 1172 system (Bruker microCT, Kontich, Belgium). At each harvest time, three cylindrical samples were obtained from the core and the cortex tissue of a single fruit, using a cork borer with 6 mm inside diameter perpendicular to the apple surface. When the fruit were large enough, after 7 weeks, cortex tissue cylinders were cut at 5 mm from the skin, core samples at 1 mm from the core line connecting the primary vascular bundles. Before week 7, the fruit were too small, and the core and cortex tissues were imaged simultaneously. Samples were wrapped in parafilm to prevent dehydration during the scan, and placed in a styrofoam holder on the rotation stage for a 24 min 3D scan at 4.8 μm image pixel resolution. The image acquisition and reconstruction protocol were adopted from Herremans et al. (2013a).

For intact fruit we used a Microfocus Computer Tomography AEA Tomohawk system (Nikon metrology160 Xi Gun set, Nikon Metrology, Heverlee, Belgium) in which larger samples could be imaged. Because the increasing size of the growing apples directly affects the achievable magnification, intact fruit were imaged at pixel resolutions from 17.4 μm on the first 5 weeks, gradually increasing to 104 μm per pixel for full grown apples. After imaging, the fruit were cut to check for the presence of internal apple disorders that might affect the internal structures, such as watercore, and discarded.

In addition, as a reference method for vasculature analysis, high-resolution X-ray CT images of intact apples at week 12, 16, and 20 were obtained using the HECTOR system (Dierick et al., 2013) at the Centre for X-ray Tomography of the Ghent University (UGCT, Gent, Belgium). The image pixel resolutions obtained were 21.2, 31.2, and 47.3 μm for the respective harvest times. The datasets were digitized as 16-bit (2000 × 2000 × 1600–1800) image stacks.

### Image processing

The X-ray CT images were reconstructed using NRecon 1.6.2.0 (Bruker microCT, Kontich, Belgium) with a filtered back projection algorithm. Three-dimensional stacks of several hundreds 8-bit gray scale images were thus generated, constituting the entire scanned volume. For the tissue sample scans, image processing involved filtering, segmentation by pixel intensity thresholding (Otsu, 1979) and removal of noise and broken objects. A 3D analysis of the microstructures was performed using CTAn 1.12.0.0 (Bruker microCT, Kontich, Belgium) to obtain binary images of intercellular air spaces and cells. Individual cells were separated and measured using the method of Herremans et al. (2013b). A brief overview of the measured morphometric parameters is given in Table 1.

**Table 1:** **Description of 3D microstructural parameters used to quantify the microstructure of apple tissue**.

**Microstructural parameter**	**Unit**	**Description**
Porosity	%	Entire void volume divided by the total volume of the analyzed sample
Number of voids per mm^3^	(mm^−3^)	Number of voids divided by the total volume of the analyzed sample
Fragmentation index	(mm^−1^)	Inverse index of connectivity, calculated as Hahn et al. (1992). A lower fragmentation signifies the presence of more concave structures, which indicates better connected material, opposed to convex structures that indicate isolated, disconnected structures
Void sphericity	(–)	Measure of how spherical an object is, defined by $\frac{{\sqrt[{3}]{\pi }\;\left(6\;\times Volume\right)}^{\frac{2}{3}}}{Surface}$, or the ratio of the surface area of a sphere with the same volume as the void to the surface area of the void
Anisotropy factor	(0–1)	Measure of preferential alignment of structures, scaled from 0 for total isotropy to 1 for total anisotropy (Odgaard, 1997)
Average void diameter	(mm)	Average of the local thicknesses of the void space or cell architecture, calculated by a skeletonization of the binarized tissue followed by a sphere fitting algorithm for each voxel of the skeleton (Hildebrand and Ruegsegger, 1997)
Equivalent cell diameter	(mm)	Average value of the cell volume, expressed as the equivalent diameter of a sphere with the same volume. To differentiate neighboring cells in the binary image, we optimized a specialized image processing tool called watershed separation, particularly suited for separating touching, convex features (Russ, 2005)

**Figure 2:**
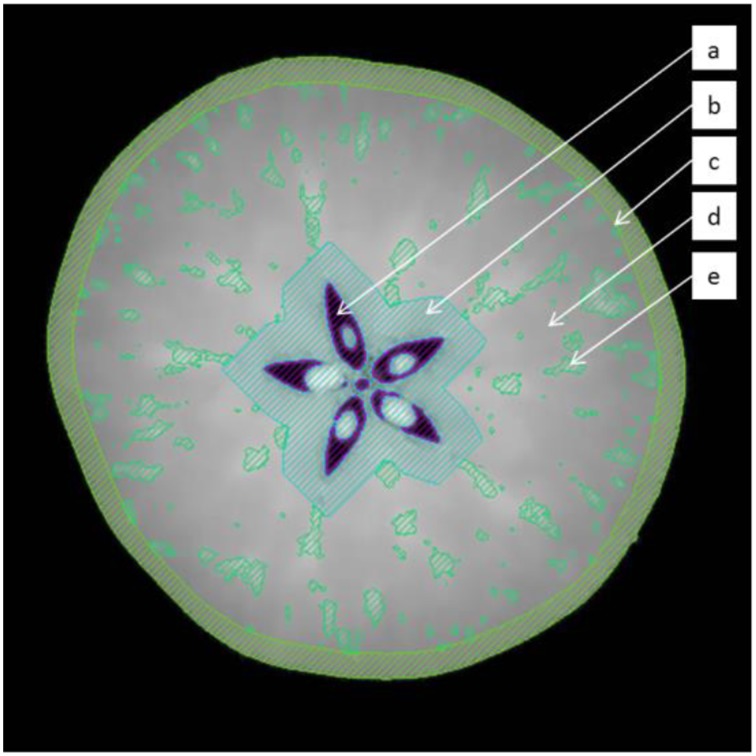
**Segmentation of the fruit in distinct regions by the image processing protocol to extract the vascular system: (a) air in the core (b) core region (c) epidermis (d) cortex (e) segmented vasculature**.

Intact apple scans were processed using Avizo Fire 8.0.1 software (VSG, Bordeaux, France). The images were filtered using an edge-preserving-smoothing filter in 3D, which is essentially a Gaussian filter that is stopped in the vicinity of certain local intensity changes such as feature edges or vascular bundles, which are of particular interest in the study. These filtered images were segmented into approximate anatomical domains, tailored to each individual fruit, using morphological operations (Figure 2). First, the air in the core was segmented by applying an intensity threshold in the obvious valley between air and apple pixels in the intensity histogram. This volume was dilated in 3D by 20 pixels, to avoid interference of the seeds and high intensity pixels in the core that interfered with the correct identification of the cortical vascular network. The entire fruit was segmented from the background, again by an obvious transition in pixel intensity between the air surrounding the fruit and the fruit itself. This volume was eroded by 14 pixels to exclude the skin and the outer, tight cell layers, as these would hinder later image processing. Then, the vascular network was segmented by means of a top hat filter with a structuring element of 4 pixels (width of the hat), and intensity threshold (height of top hat) value of 4 (Russ, 2005). This segmented network was skeletonized (Avizo Fire 8.0.1) in order to extract the essential vasculature topology, i.e., a point cloud with connectivity information between different points and local thickness measures of the vessels. The image processing protocol was validated by manually segmenting vascular bundles in high-resolution datasets of apple fruit. This is not an absolute validation either, but in the absence of a “gold standard,” it is the best available 3D representation of the vascular network (Walter et al., 2010). A region-growing algorithm was used to manually segment the vascular network. This algorithm selects pixels of a certain (high) intensity that are connected in 3D to a manually picked seed pixel. Visual inspection by matching the selected regions to the high intensity pixels in the reconstructed slices, and making sure that the selection in 3D resembles tube-like structures, ensured that the selected structures correspond to vascular bundles.

The branching points of the vascular networks were isolated and plotted in terms of the radial distance in the fruit using Matlab 2013 (Mathworks, Inc., Natick, MA) to express the spatial connectivity of the network. These processed images enable the quantification of the tissue structures by extracting morphological figures that were compared statistically by performing Analysis of Variance (ANOVA) and Tukey's test (*P* < 0.05) using JMP 11 (SAS Institute Inc., Cary, NC).

## Results

### Development of fruit weight, size, and firmness

**Figure 3:**
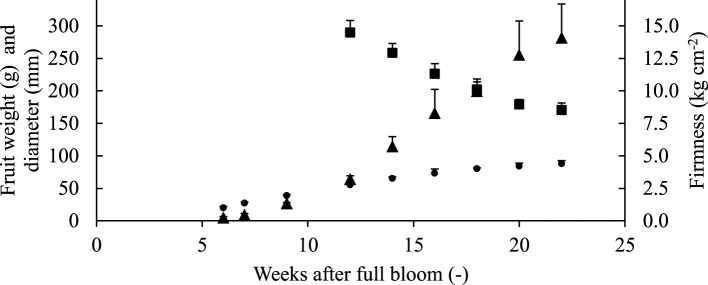
**Evolution of fruit fresh weight ▴(g), equatorial diameter ● (mm), and firmness ■ (average firmness on the red side) of “Jonagold” apples of the same orchard**. Error bars are standard deviation.

Throughout the growing season, the diameter and weight of the fruit increased steadily (Figure 3). The average fruit firmness decreased to values of 7–8 kg cm^−2^ that are typical for mature “Jonagold” fruit (Schenk, 2013).

### Development of air voids in apple fruit

**Figure 4:**
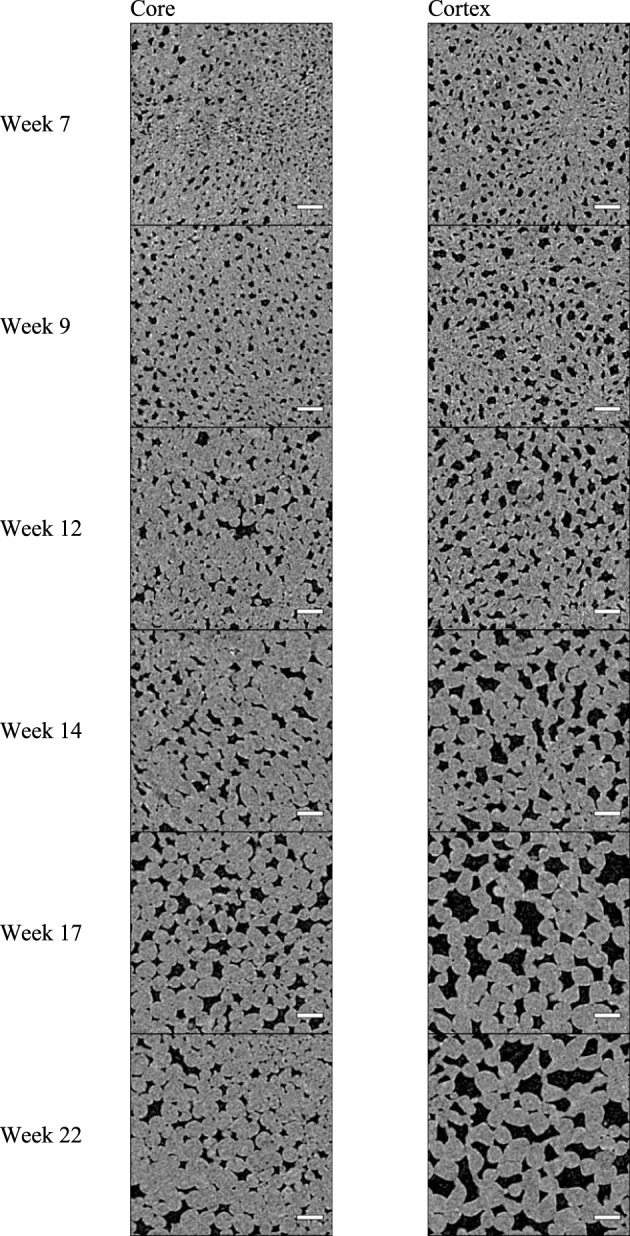
**X-ray CT virtual cross-sections through “Jonagold” core and cortex tissue at different times during apple fruit development: 7, 9, 12, 14, 17, and 22 weeks after full bloom), obtained by means of the SkyScan 1172 system**. Scale bars indicate 250 μm. Images measure 1.9 × 1.9 mm.

Two-weekly scans of the apple parenchyma samples revealed considerable changes in the microstructure of the core and cortex (Figure 4). The black objects are the gas-filled voids which are located at many positions, intertwined between the cells in such a way that a porous tissue is formed. With time proceeding and fruit developing on the tree, the cells and the voids clearly increased in size. Based on these 2D cross-sections the voids in the core appeared smaller than those in the cortex.

**Figure 5:**
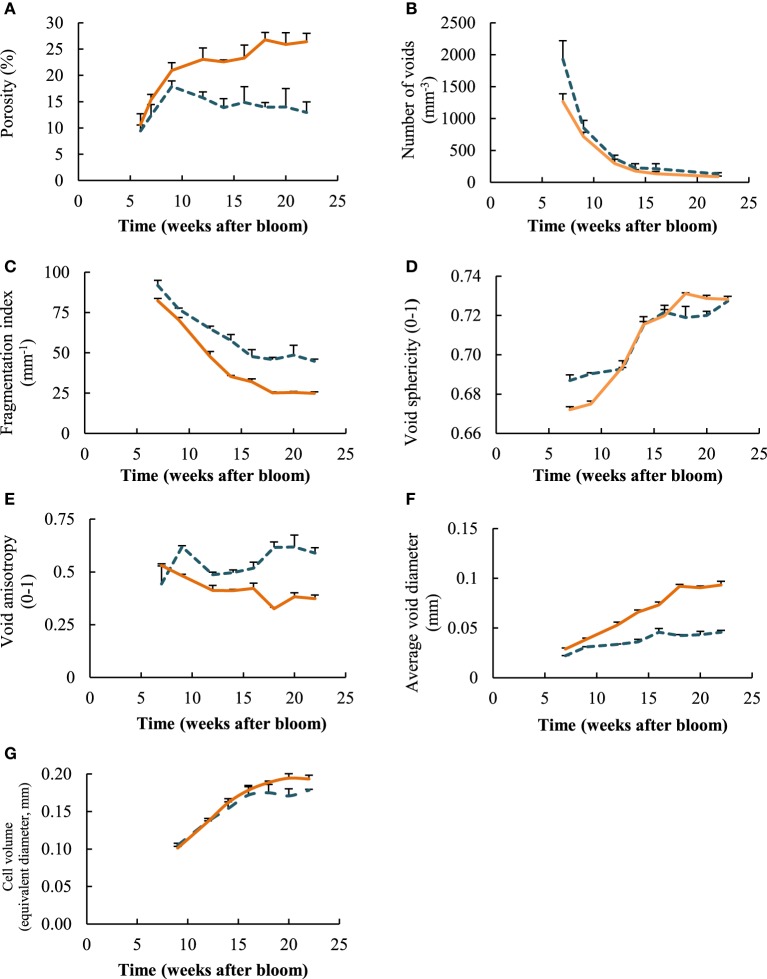
**Evoluation of microstructural parameters for core (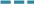) and cortex (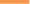) tissue samples as a function of weeks after bloom: (A) porosity, (B) number of voids, (C) fragmentation index, (D) void sphericity, (E) void anisotropy, (F) average void diameter, (G) equivalent diameter of cells**. Error bars represent the standard error of 3 independent measurements.

Microstructural parameters from the 3D images of the different time steps and sample locations showed that overall presence of voids, represented by the porosity of the tissue, increased significantly for the measured cortex samples (Figure 5A), from 10.6% at week 7 to 26.4% in week 22. The porosity leveled off from week 14 onwards. The average porosity of the core tissue was significantly lower than that of the cortex, except for the earliest measurement session in week 7. The porosity in the core did not change significantly during the entire growth season. Overall, the porosity of the core was equal to 14.5% on average.

The number of individual voids in a specific volume decreased tremendously during the growing season, for both core and cortex samples (Figure 5B). The measured core samples contained more than 1900 (± 294) voids per mm^3^ at start of growth, and cortex samples more than 1250 (± 122) voids per mm^3^. At the time of optimal harvest, these numbers dropped significantly to as low as 132 (± 18) and 88 (±10) voids per mm^3^ for core and cortex tissue, respectively. This can be partly attributed to the average increase in volume of the voids, as well as the cells for that matter, in such a way that fewer voids fit in the same volume of the tissue. However, there was also an observed significant change in fragmentation (Figure 5C) during growth that was similar for the core and cortex tissue samples. The fragmentation of the void space decreased consistently during fruit development, indicating the void space networks became increasingly connected. The void space of the core remained on average more fragmented at the end of growth season (Figure 5C), which is in agreement with the earlier stop in the increase of core porosity compared to the observations in the cortex. The shape of the voids, expressed as the sphericity (Figure 5D), rose during the season, as the volume to surface ratio of the voids changed, and the voids became more spherical. The void space in the core was significantly more anisotropic than those of the cortex (Figure 5E). This means the voids have a more distinct orientation in the core tissue, forming pathways in primarily radial directions.

Comparing local diameters of the void structures (Figure 5F), it is clear that the average void diameter increased, with the cortex tissue having larger voids than the core at the end of fruit development. The average local diameter of the void network increased from 28.8 ± 3.2 to 93.2 ± 11.7 μm. Although larger single void structures were found in the core (Figure 4), in general their diameters remained smaller throughout the growth: from 22.15 ± 0.49 μm at 7 weeks after bloom to 45.8 ± 5.9 μm at harvest. Interestingly, the average individual cell sizes (Figure 5G), do not differ significantly between the core and the cortex samples. In contrast to the voids, the cells in these tissues with different anatomical origins, reached similar sizes while growing.

**Figure 6:**
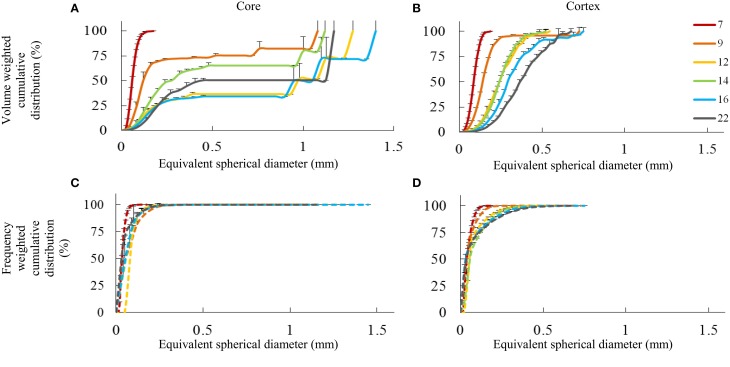
**Pore space distribution expressed as cumulative volume fraction (A,B) and cumulative frequency fraction (C,D) of individual pores as a function of their equivalent diameter (mm) for core and cortex tissue, measured at several weeks after bloom (color legend)**. Error bars represent the standard error of 3 independent measurements.

Evolution in void sizes and shapes is also reflected by the distribution of void volumes (Figure 6). For both core and cortex samples, there was an observed shift toward larger void volumes (expressed as the equivalent spherical diameter of individual voids) in terms of the volume weighted void distribution as the fruit developed. Over time, there was a gradual decrease in small volumes, followed by an absence of voids with equivalent diameter roughly between 0.5 and 1 mm, and then finally the appearance of larger, single voids that comprise a significant volume of the airspace. In particular in the core samples (Figure 6A), sudden jumps in the volume distributions indicate the presence of highly connected networks of voids.

In cortex samples (Figure 6B), the volume weighted cumulative distribution of the equivalent void diameter shifted to the right as the fruit developed. However, the void volumes were more evenly distributed than in the core without discrete jumps, and the largest, continuous void volumes (0.75 mm spherical diameter) were smaller than in the core (1.4 mm spherical diameter). As the fruit grew, the void volumes thus grew as well. Single, connected void networks comprising a quarter to half of the total void volume were not present in the cortex. There was no direct indication that new voids were formed during fruit development after 7 weeks after full bloom, but there is certainly a significant change in the configuration of the void network.

Comparing volume weighted distributions to frequency weighted (Figures 6C,D), it appears that in terms of numbers, the majority of the voids were quite small, but they contributed only modestly to the total void volume. As the fruit develops, more voids of larger size appear; however, the smaller pores remain dominant in terms of numbers. In the core in particular, only a few, highly connected, large void networks comprise the majority of the void volume.

### Development of vascular bundles in apple fruit

**Figure 7:**
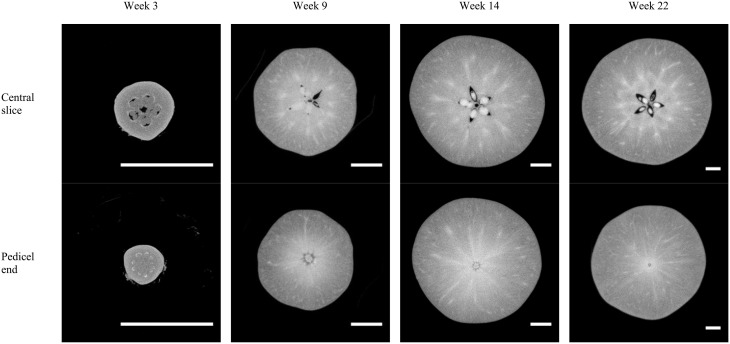
**Examples of X-ray micro-CT cross-sections of “Jonagold” apple fruit at different time steps during fruit development, obtained by means of the Skyscan system (week 3) or Tomohawk system (weeks 9, 14, and 22)**. The scale bars measure 1 cm.

X-ray micro-CT cross-sections (Figure 7) of the entire fruit resolved internal structures such as the air in the core and the ovules or seeds. The vascular bundles are shown throughout the development stages of the fruit as spots with high intensity, i.e., bright pixels. This indicates a higher attenuation of the X-rays, caused by a dense tissue microstructure. The latter is likely due to the relatively thick vessel structures, water within the vessels, and compact phloem with companion and parenchyma cells surrounding the xylem.

**Figure 8:**
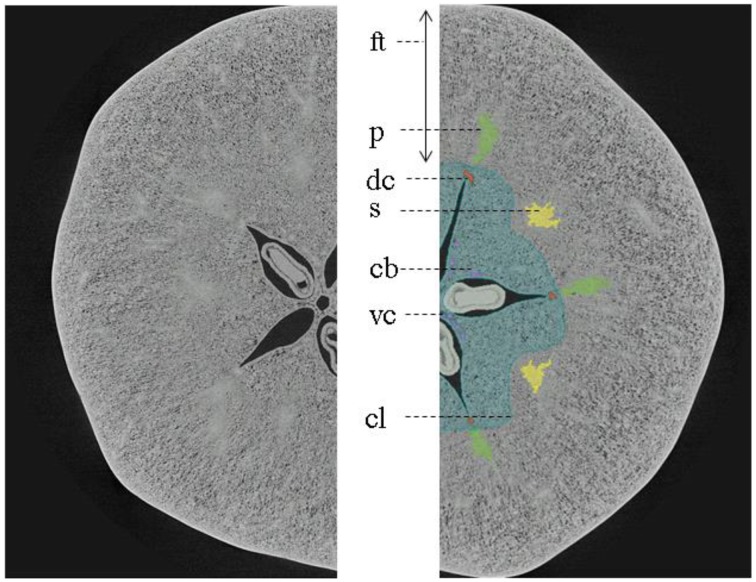
**X-ray micro-CT cross-section of “Jonagold” apple fruit harvested after 12 weeks after bloom showing gross morphology ft, floral tube; s (yellow), sepal bundles; p (green), petal bundles; cl (blue), outer limit of carpel or core line; dc (orange), dorsal carpellary bundle; vc (pink), ventral carpellary bundle; and cb, carpellary bundles connecting dorsal with ventral carpellary bundles**. The image was obtained by means of UGCT's HECTOR system (Dierick et al., 2013).

**Figure 9:**
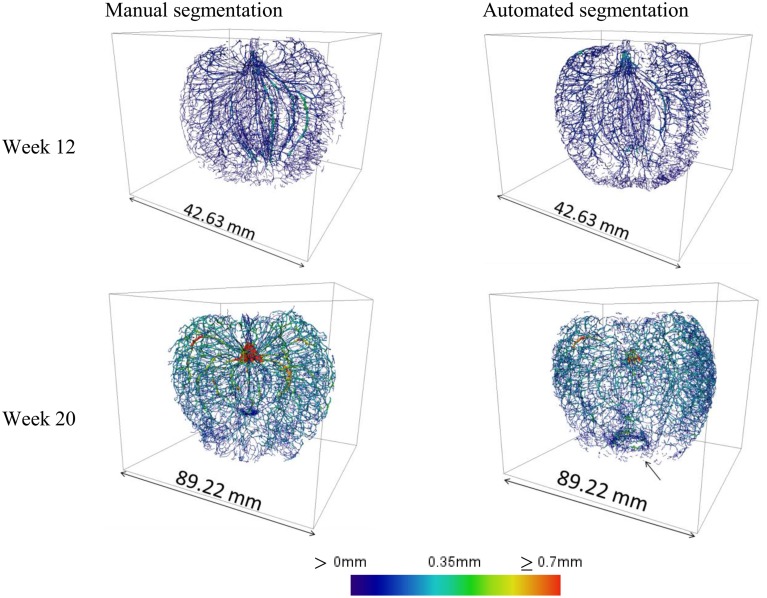
**Cortical vascular networks of apple fruit of weeks 12 and 20, obtained by means of manual image processing of high-resolution datasets and by application of the automated image processing protocol**. The arrow indicates a ring that is an artifact created by the skeletonization procedure where the vascular bundles merge. At week 12 the total vasculature length was 7.07 and 6.17 m manual and automated segmentation, respectively; at week 20 this was increased to 18.81 and 17.06 m, respectively.

The main vascular structures can be readily resolved in the high-resolution images, showing the cortical and carpellary vascular systems (Figure 8). The automatically and manually segmented vascular networks of the same apples are shown in Figure 9, showing the cortical bundles only. The main differences are in the smaller ramifications and local thickness measurements. In the automated image processing, there is an underestimation of about 10% of the total vasculature length compared to that of the manually segmented networks.

Also, at the calyx end, an annular artifact was produced in some datasets due to the vascular bundles that merge. However, the automated processing took only a fraction of time needed for the manual work (15 min compared to over 10 h), and overall, gave a satisfactory first impression of the global vasculature structure.

**Figure 10:**
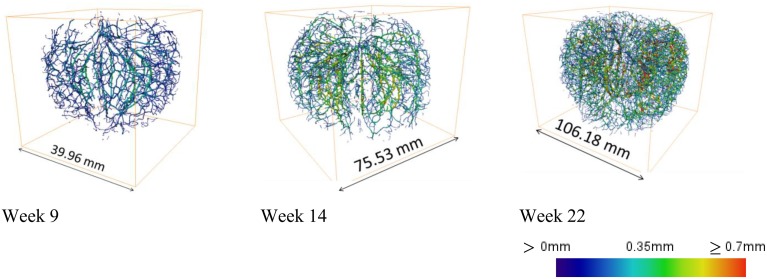
**Examples of 3D cortical vascular networks obtained by means of the automated image processing protocol at 9, 14, and 22 weeks after bloom using Tomohawk datasets**. Colors indicate thickness of the vasculature, calculated as the local radius of the vascular bundles, expressed on a scale between 0 and 0.7 mm.

Automated image processing of datasets obtained on intact apple from week 9 onwards, created 3D vasculature maps of developing fruit (Figure 10). Due to insufficient image contrast to resolve the vascular bundles in tissue with a low porosity, the automated procedure could not be applied to younger fruit. This presents also a potential limitation of the method for application to other fleshy fruit with limited porosity. Other solutions including the use of X-ray contrast agents could then be considered.

The resulting networks offer information of local radii of the vasculature and the connectivity of the bundles by branching. The main cortical bundles were on average thicker than the vascular bundles that branch toward the skin as indicated by the color scale.

**Figure 11:**
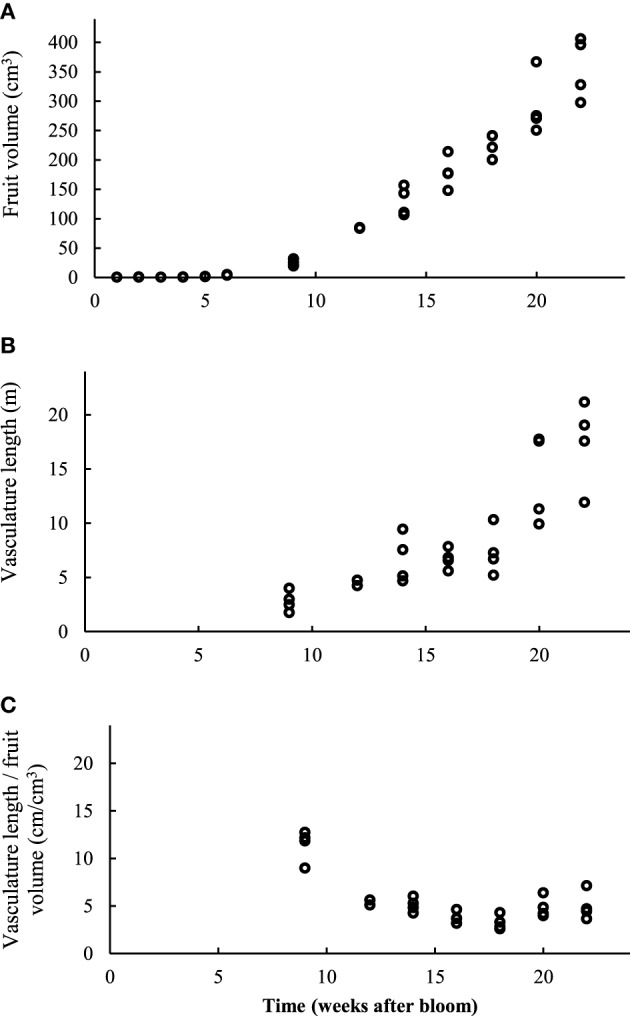
**(A)** Evolution of the total fruit volume, determined by 3D measurement of the image data stack. **(B)** Total length (m) of the automatically segmented vascular network in intact apples at different times. **(C)** Total vasculature length (cm) divided by the intact apple volume (cm^3^) at different harvest times (4 replicates).

Also as growth progressed, the vascular bundles grew, both in terms of average diameter as well as lengthwise (Figure 11). At week 9, the length of the vascular bundles was 2.81 (± 0.95) m, at week 14, 6.7 (± 2.22) m, and at week 22, as much as 17.42 (± 3.96) m. Surprisingly, the length of the vascular bundles expressed on a fruit volume basis did not change significantly from 12 weeks after bloom onwards, and leveled out at around 5 cm per cm^3^ of fruit volume. Although this value seemed to rise slightly in the end again, this trend was not significant. Young developing fruit had a more extensive vascular network with respect to their rather small volume (11.42 cm bundle length per cm^3^ of apple volume). The fruit initially appeared to grow faster than the vascular network. However, toward the end of the fruit development the vascular network seemed to catch up after a period of relative slow growth. It also seems that the vascular bundles at the pedicel end of the fruit were more developed than at the calyx end.

**Figure 12:**
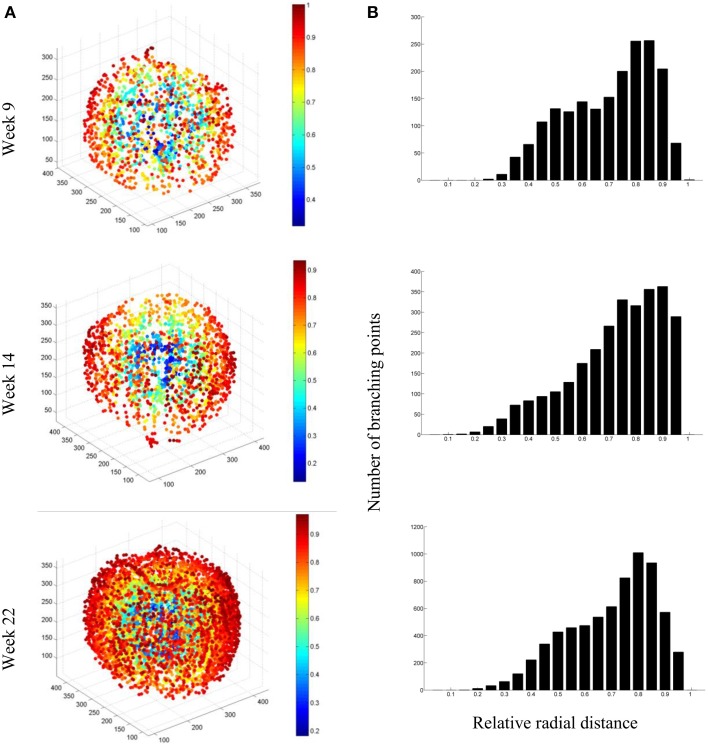
**(A)** Examples of network connectivity plots of the segmented vascular networks of fruit at 9, 14, and 22 weeks after bloom, showing the branching points and their radial position in the fruit (color bar). **(B)** Distribution of branching points as a function of radial distance from the core of the fruit.

To quantify the evolution of branching of the vascular network, which seems to increase substantially by visually evaluating the vascular networks, we isolated the branching points (vertices) in the network, and plotted them as a function of the relative radial position in the apple (Figure 12). The increase in vasculature length at the end of the growth season appeared to be largely due to increased branching near the skin: from week 14 to 22, the number of vertices at radial distances up to 0.5*R* approximately doubled, with *R* the radius of the fruit; above 0.8*R* the number increased four-fold. One can also calculate the vessel density as a function of radius (not shown). In mature fruit, the vessel density peaked at about 20 mm (7.5 cm per cm^−3^) from the center where the main bundles are situated. In the cortex, the vessel density increased from 3 at 25 mm from the center to 5 cm per cm^−3^ near the surface.

## Discussion

### Implications of the distinct void architecture in core and cortex tissue

As of week 7 after full bloom and later, the organization of voids in the core and cortex tissue appeared to be different: the porosity of the core was smaller than that of the cortex, and the void network in the core was more narrow, branched and fragmented. This most likely has an impact on transport of respiratory gasses (Ho et al., 2011, 2013; Herremans et al., 2013a, 2014a,b). The diffusivity of metabolic gasses such as O_2_ and CO_2_ is highly dependent on the porosity and connectivity of the cortex tissue (Ho et al., 2011). It has been shown that a low diffusivity for metabolic gasses in combination with a high respiratory activity may cause hypoxic conditions in the center of apple fruit that may lead to cell death and visible tissue damage symptoms including browning and the emergence of internal cavities. This has serious consequences for the commercial storage of apple fruit which commonly is under hypoxic conditions.

The increase in individual pore volume in the cortex during fruit development may impart sponginess to the texture of apple fruit and contribute to the characteristic reduction of firmness that is typically observed before harvest (Figure 3). However, the increase in porosity levels off in the final weeks before harvest, whereas the firmness continues to decrease. This can be attributed to changes in the cell wall and, in particular, middle lamella biochemistry that inflicts further textural changes as the fruit matures (Johnston et al., 2010; Longhi et al., 2012; Gwanpua et al., 2014). The gradual loosening of the cells due to pectin breakdown, particularly at positions were three or more cells touch each other, may create little schizogenous pores that are likely not to increase the porosity but may further increase the connectivity of the pore network. We have observed such schizogenous pores in apple cortex tissue based on high resolution synchrotron μCT images (Verboven et al., 2008); in pear cortex tissue they are abundant.

Recent advances in X-ray μCT hardware, software and computational power (Hanke et al., 2008; Pratx and Xing, 2011) allows to visualize relatively large samples (such as intact apple fruit in Figure 8) at imaging resolutions that allow microstructural assessment, such that the entire void space of the tissue can be imaged for an entire fruit. These huge data stacks enable the generation of a virtual fruit geometry combining detailed information from the fruit scale to the void scale, based on measurements that display genuine heterogeneities in the fruit microstructure, without the need for sampling. Such high resolution images will provide much more detail about gas transport processes in apple fruit.

### Development of voids suggest lysigenous origin

The smallest voids we could identify in apple tissue measured 3.2 × 10^−6^ mm^3^. This lower limit is due to the imaging resolution window of the used X-ray micro-CT technology. This implies that the small pores early in the growth phase were beyond the resolution of the μCT platform we used. With the applied protocol, we could resolve pores in the tissue starting at 7 weeks after bloom using X-ray CT, not sooner. Other imaging technologies are probably more suited to obtain high resolution images at high magnifications such as electron microscopy to study the early stages of pore formation; however, this is at the expense of obtaining 3D volume image stacks unless more advanced electron tomography techniques are used. Also, such techniques often require sample preprocessing (such as critical point drying) that may introduce artifacts.

The void networks in the core are significantly narrower than the voids in the cortex. In both the cortex and core tissue, the voids have similar diameters as the cells and thus suggest a lysigenous origin. They are thus likely a consequence of programmed cell death (PCD), possibly initiated by low oxygen conditions in the center of the fruit when its diameter and thus gas diffusion resistance is increasing during growth. PCD creating aerenchyma in water logged plants involves ethylene and calcium, while changes in local ROS production might also play a role under low oxygen conditions in e.g., maize roots (Evans, 2003; Verboven et al., 2012; Yamauchi et al., 2013; Van Hautegem et al., 2014; Broughton et al., 2015). PCD has been observed in fruit under external stress (Chen et al., 2014; Zheng et al., 2014) but evidence of the phenomenon has not yet been given for pore formation during fruit growth. To our knowledge no studies have yet been conducted on PCD during growth of fruit organs.

After 9 weeks the frequency weighted pore distribution and the porosity of both the core and the cortex does not change much anymore; but the pore size keeps increasing. This indicates that lysigenous pore formation essentially happens during the cell division developmental phase which was most likely finished when this experiment started; during the subsequent cell enlargement development phase pores essentially grow in line with the cells. Any additional pore formation during this development stage is likely schizogenous and caused by middle lamella separation. High resolution imaging of the cell division developmental phase is required to further detail pore formation in apple fruit.

As a result of different mechanisms of void formation, voids will have different sizes, shapes and connectivity. This will affect the gas diffusion properties of the tissue (Ho et al., 2011; Verboven et al., 2014) that consequently affects respiratory processes in the bulky organs. A better understanding of void formation and characteristics thus will assist knowledge and control of postharvest physiology of the fruit (Franck et al., 2007).

### Vascular network continues to extend from main vessels

X-ray micro-CT provided a 3D visualization of the vascular system in apple fruit. From the images, it was found that the total length of the vascular system can be as much as 20 m. This vascular system was identified through connected regions in the CT images with local peaks in pixel intensity indicating the near absence of pores. However, the resolution of the obtained X-ray CT images is not suited for direct visualization of the xylem and phloem. We thus essentially visualized the tightly packed cell configuration surrounding the vessels. In this regard, the local vasculature thickness must thus be interpreted as that of the entire vascular system including its immediate surrounding parenchyma tissue. Consequently, information about local disruptions or changes in the xylem or phloem conductivity cannot be obtained. As MRI traces the water filled vessels, it suffers from similar issues of detectability of the vascular tissue (Moriwaki et al., 2014). The recent results obtained with MRI of a single and smaller apple of a different cultivar (Moriwaki et al., 2014), however, provide little basis for comparison of micro-CT and MRI. The MRI results present only the vascular volume that increases with growing season in a similar fashion to vascular length provided here. Our results also provide the vessel vascular diameter that varies considerably from the center to the surface of the fruit. In addition, we quantified the spatial variation of the branching structure. Other quantities that we obtained but not reported and discussed here are the length and direction of individual vascular segments between branching points. Together, these measures quantitatively and comprehensively describe the 3D vascular system layout in the fruit.

Pollination stimulates vascular development and differentiation for the development of the pistil and hypanthium into a fruit (Gillaspy et al., 1993; Tiwari et al., 2013). Influencing factors include auxin, ethylene, assimilate availability, assimilate utilization, and dominance of competing fruit, and external factors such as heat stress (Tiwari et al., 2013). It has been demonstrated in apple that an auxin-like signal emanating from the fruit not only stimulates vessel differentiation in the pedicel but also controls fruit and seed development (Drazeta et al., 2004a). Formation and growth of vascular bundles is associated with inhomogeneous auxin distribution patterns mediated by efflux carrier proteins (Benková et al., 2003; Van Hautegem et al., 2014), for which the mechanisms are only starting to be described (Merks et al., 2011; De Vos et al., 2012; Hayakawa et al., 2015).

In apple, phloem and xylem flows contribute almost equally to fruit growth during the first part of the season as the xylem provides a path to supply the water and minerals while the phloem delivers transport sugars to the growing cells. However, as fruit develop, the contribution of xylem flow is progressively reduced, and around 90 days after full bloom apple growth is sustained only by the phloem (Lang, 1990). Most likely, due to the expansive growth of the tissue, the xylem vessels experience excessive stresses and are broken, thus becoming dysfunctional for transport (Drazeta et al., 2004c). This is evident in the changes in the shape of the vascular network, and the observed expansive growth of the cells and voids which causes the typical apple shape with its indented calyx and stem ends. While breakages of the xylem bundles were not observed at the used imaging resolution, the study observed continuous growth and branching of the vascular system throughout fruit growth and development. This may be attributed to the phloem that consists of living parenchyma cells that are more flexible to sustain growing tissue and possibly differentiate and even divide to form new phloem sieve cells and companion cells, even after the main cell division stage has finished, maintaining the transport of solutes in growing fruit. The signals that trigger these prolonged divisions are not known yet. Considering the fact that we measured 5 cm of vasculature per cm^3^ of tissue, there might be an optimal cell to vascular bundle distance involved. The images confirm that the vascular bundles form an intricate 3D network that pervases the whole fruit during the developmental stage (fruit maturation; cell expansion developmental phase) that was considered here. They can be used to validate growth models for vascular systems.

Using the 3D vasculature wiring diagrams of the apple, it is now possible to model water and nutrient flow in the fruit as well as investigate the occurrence of the mineral imbalances, and thereby explain and predict the typical appearance of certain disorders in the fruit (Lang, 1990). An example of the latter is bitter pit, which is connected with an irregular water supply and shifts in the local calcium content, an element that is exclusively transported through the xylem. The regions that are farthest away from the supply through the pedicel, are more likely to become depleted, and are therefore, more susceptible to bitter pit (Ferguson et al., 1999).

Xylem hydraulics have been recently studied in *A. thaliana* to understand the functional traits of xylem (such as growth rate, resistance to drought), and link these to certain genotypes (Tixier et al., 2013). In a broader context, understanding the water economics of plant systems is of great concern in a changing environment (McDowell et al., 2008). Understanding this long-distance water transport in plants requires, among other, the integration of hydraulics with anatomy and ecology (Tixier et al., 2013). By connecting vasculature models of individual apples to previously constructed hydraulic models for wood (Brodersen et al., 2011), root (Wu et al., 2011), and leaf (Blonder et al., 2012), a virtual tree could be generated, with sap flows from the soil toward leaves and fruit. In order to use the present results in a modeling framework, X-ray tomography of intact fruit should be combined with a histological study of the same fruits (if possible) in order to assess, at different locations, the diameter of xylem and phloem vessels, which could not be distinguished here. This could be achieved with conventional microscopy methods or with high resolution X-ray microtomography that can resolve the different cell types in the vasculature (Verboven et al., 2014).

## Author contributions

EH developed the imaging and analysis protocols and drafted the manuscript. EH and TD carried out the X-ray CT measurements. MV and DC participated in image processing. MH assisted in data analysis and interpretation. PV supervised the study, participated in its design and coordination, and helped to draft the manuscript. LV and BN initiated and coordinated the study. All authors read and approved the final manuscript.

### Conflict of interest statement

The authors declare that the research was conducted in the absence of any commercial or financial relationships that could be construed as a potential conflict of interest.
